# Spatiotemporal and genomic analysis of carbapenem resistance elements in Enterobacterales from hospital inpatients and natural water ecosystems of an Irish city

**DOI:** 10.1128/spectrum.00904-24

**Published:** 2024-11-27

**Authors:** Mark Maguire, Carlos Serna, Natalia Montero Serra, Aneta Kovarova, Louise O’Connor, Niamh Cahill, Brigid Hooban, Niall DeLappe, Wendy Brennan, Genevieve Devane, Martin Cormican, Dearbháile Morris, Simone C. Coughlan, Georgios Miliotis, Bruno Gonzalez-Zorn, Liam P. Burke

**Affiliations:** 1 Antimicrobial Resistance and Microbial Ecology Group, School of Medicine, University of Galway, Galway, Ireland; 2 Center for One Health, Ryan Institute, University of Galway, Galway, Ireland; 3 SFI Center for Research Training in Genomics Data Science, Dublin, Ireland; 4 Antimicrobial Resistance Unit, Animal Health Department, Faculty of Veterinary Medicine, Complutense University of Madrid, Madrid, Spain; 5 National Carbapenemase Producing Enterobacterales Reference Laboratory Service, University Hospital Galway, Galway, Ireland; University of Pittsburgh School of Medicine, Pittsburgh, Pennsylvania, USA

**Keywords:** antibiotic resistance, plasmids, transposons, Enterobacterales, carbapenems, environmental microbiology

## Abstract

Carbapenemase-producing Enterobacterales (CPE) is a diverse group of often multidrug-resistant organisms. Surveillance and control of infections are complicated due to the inter-species spread of carbapenemase-encoding genes (CEGs) on mobile genetic elements (MGEs), including plasmids and transposons. Due to wastewater discharges, urban water ecosystems represent a known reservoir of CPE. However, the dynamics of carbapenemase-bearing MGE dissemination between Enterobacterales in humans and environmental waters are poorly understood. We carried out whole-genome sequencing, combining short- and long-sequencing reads to enable complete characterization of CPE isolated from patients, wastewaters, and natural waters between 2018 and 2020 in Galway, Ireland. Isolates were selected based on their carriage of Class A *bla*_KPC-2_ (*n* = 6), Class B *bla*_NDM-5_ (*n* = 12), and Class D *bla*_OXA-48_ (*n* = 21) CEGs. CEGs were plasmid-borne in all but two isolates. OXA-48 dissemination was associated with a 64 kb IncL plasmid (62%), in a broad range of Enterobacterales isolates from both niches. Conversely, *bla*_KPC-2_ and *bla*_NDM-5_ genes were usually carried on larger and more variable multireplicon IncF plasmids in *Klebsiella pneumoniae* and *Escherichia coli*, respectively. In every isolate, each CEG was surrounded by a gene-specific common genetic environment which constituted part, or all, of a transposable element that was present in both plasmids and the bacterial chromosome. Transposons Tn*1999* and Tn*4401* were associated with *bla*_OXA-48_ and *bla*_KPC-2_, respectively, while *bla*_NDM-5_ was associated with variable IS*26* bound composite transposons, usually containing a class 1 integron.

**IMPORTANCE:**

Since 2018, the Irish National Carbapenemase-Producing Enterobacterales (CPE) Reference Laboratory Service at University Hospital Galway has performed whole-genome sequencing on suspected and confirmed CPE from clinical specimens as well as patient and environmental screening isolates. Understanding the dynamics of CPE and carbapenemase-encoding gene encoding mobile genetic element (MGE) flux between human and environmental reservoirs is important for One Health surveillance of these priority organisms. We employed hybrid assembly approaches for improved resolution of CPE genomic surveillance, typing, and plasmid characterization. We analyzed a diverse collection of human (*n* = 17) and environmental isolates (*n* = 22) and found common MGE across multiple species and in different ecological niches. The conjugation ability and frequency of a subset of these plasmids were demonstrated to be affected by the presence or absence of necessary conjugation genes and by plasmid size. We characterize several MGE at play in the local dissemination of carbapenemase genes. This may facilitate their future detection in the clinical laboratory.

## INTRODUCTION

Antimicrobial resistance (AMR) is a major public health threat, recognized by the scientific community, the World Health Organization, and national governments (1, 2). β-lactams (penicillins, cephalosporins, and carbapenems) are the most frequently used antibacterial agents due to their wide activity spectra, high bactericidal efficacy, and nearly optimal selective toxicity (3, 4). The primary mechanism of β-lactam resistance in Gram-negative bacteria is the production of β-lactamases, i.e., enzymes hydrolyzing the amide bond of the β-lactam ring.

Carbapenems, including meropenem, imipenem, doripenem, and ertapenem, are used as critically important reserve antibiotic therapies when other more commonly used agents are not appropriate. The importance of these agents is reflected in high mortality rates (30%–75%) among patients with invasive carbapenem-resistant Enterobacterales (CRE) infections (5).

The CRE phenotype predominantly arises from the production of various acquired β-lactamases with wide substrate spectra, the most important of which are *Klebsiella pneumoniae* carbapenemase (KPC), oxacillinase (OXA), New Delhi metallo-β-lactamase (NDM), Verona integron-encoded metallo-β-lactamase (VIM), and imipenemase (IMP) types. They are collectively called “carbapenemases,” which reflect the clinical impact of these enzymes rather than any close phylogenetic associations or similarities in structural-functional traits. Carbapenemases are distributed into three of the four classes (A, B, and D) of the Ambler molecular classification scheme for β-lactamases (6). Class A serine carbapenemases include KPC, Class B carbapenemases are metallo-β-lactamases like NDM, and Class D comprises carbapenem-hydrolyzing Class D β-lactamases such as OXA-48 (7).

Carbapenemase-encoding genes (CEGs) are carried on a range of different mobile genetic elements (MGEs), most importantly plasmids and transposons, allowing their spread both within and between many different bacterial species and genera (8). Characterization of these MGE is valuable to support understanding, monitoring, and controlling multi-species MGE-based outbreaks. At present, this is challenging for many reference laboratories. Commonly used next-generation sequencing (NGS) and analysis technologies are insufficient to facilitate complete resolution and classification of MGE present in distinct carbapenemase-producing Enterobacterales (CPE) species genomes.

CPE was declared a public health emergency in Ireland in 2017. In that context, there was significant investment in enhanced detection of CPE colonization and in infection prevention and control measures (9). Most patients admitted into acute hospitals in Ireland are offered screening for CPE carriage, preferably within 24 hours of admission, through the collection of a rectal swab or stool sample. CPE is detected by selective culture or molecular testing. The first isolate of each species of CPE from each patient is referred to the National CPE Reference Laboratory (NCPERL). NGS is carried out on all isolates, and the resulting genomes are analyzed bioinformatically to determine the core genome multi-locus sequence type (MLST), resistance genotype, and certain carbapenemase-carrying plasmids (10).

The number of newly detected patients with CPE has shown an upward trend since 2013. This is true of diagnostic samples and surveillance samples. The most commonly detected CEG in Ireland to date is *bla*_OXA-48-like_ (73%), followed by *bla*_NDM_ (9%) and *bla*_KPC_ (8%) (11). Detection of other carbapenemase genes and detection of more than one such gene in a single isolate are still relatively infrequent (12). The importance of environmental reservoirs in the hospital has also been increasingly recognized since 2018 with CPE detected at numerous hospitals especially in damp areas such as showers, sinks, and toilets (13–15).

Despite advances, knowledge gaps exist in our understanding of the role of the environment in AMR spread and in the directionality of spread from human carriage/infection to and from the environment (16). A recent Irish study investigating the prevalence of AMR in the environment over 2 years (2018–2020) collected 218 samples from both wastewaters (*n* = 61) and natural waters (*n* = 157) at various locations in Ireland. This study detected CPE at 31 (14%) sampling sites, with a total of 37 CPE isolates detected. These carried a variety of carbapenemase genes including *bla*_KPC_, *bla*_NDM_, and *bla*_OXA-48-like_ genes (17, 18). The relationship between environmental CPE isolates and those from patients and the hospital environment has not been adequately explored in Ireland.

The primary aim of this study was to characterize and genomically survey CPE isolates and their associated CEG-bearing MGEs collected between 2018 and 2020 from hospital patients, clinical and wastewater environments, and natural water environments in Galway city, Ireland. Hybrid whole-genome sequencing (WGS) analysis facilitated complete genomic characterization of CPE chromosomes and MGEs. Spatiotemporal analysis facilitated the assessment of potential dissemination of CPE clones and carbapenemase-bearing plasmids between species and ecological niches. A secondary aim of this study was to assess the transferability of several identified CEG harboring plasmids utilizing conjugation assays.

## MATERIALS AND METHODS

### Isolate selection and description of bacterial isolates

Galway city, Ireland, was chosen as the study site. Galway has a population of 84,414 (19) served by University Hospital Galway which has a bed capacity of 643 beds. A previous study by Hooban et al. (17, 18) isolated 20 CPE isolates from seawater (*n* = 12), river estuary water (*n* = 5), and sewage (*n* = 3) in Galway city, carrying *bla*_OXA-48_ (*n* = 10), *bla*_KPC-2_ (*n* = 3), and *bla*_NDM-5_ (*n* = 7; see Table S1).

To facilitate comparison, clinical isolates from the NCPERL were selected on the basis that (i) they carried the same carbapenemase genes, (ii) were isolated from hospital patients or the hospital environment in the same urban location, and (iii) within the same period of 2018–2020. A total of 18 clinical isolates were selected and were from patient rectal swabs or stool samples (*n* = 15), from blood (*n* = 2), and from the hospital environment (*n* = 1). The clinical isolates were positive for *bla*_OXA-48_ (*n* = 10), *bla*_KPC-2_ (*n* = 3), and *bla*_NDM-5_ (*n* = 5). The *bla*_KPC-2_ and *bla*_NDM-5_ isolates represented the entire set of clinical CPE isolates bearing these genes collected within the study timeframe. To select clinical isolates of bla_OXA-48_ producers, a previously described pOXA-48-like plasmid MLST approach based on 71 genes (20) was first used to classify the plasmid types from short-read assemblies of all bla_OXA-48_ producing clinical CPE isolates collected in Galway during the study period (*n* = 294). Ten clinical isolates were chosen for further analysis, based primarily on a representative diversity of plasmid types, to include several species and sequence types (STs).

### Phenotypic characterization of CPE isolates

Antimicrobial susceptibility testing was carried out on all isolates by disc diffusion according to EUCAST guidelines (21). Antimicrobial agents included ampicillin (10 µg), cefoxitin (30 µg), cefpodoxime (10 µg), cefpodoxime/clavulanic acid (10 µg/1 µg), ceftazidime (10 µg), cefotaxime (5 µg), ertapenem (10 µg), meropenem (10 µg), gentamicin (10 µg), kanamycin (30 µg), streptomycin (10 µg), tetracycline (30 µg), chloramphenicol (30 µg), nalidixic acid (30 µg), ciprofloxacin (5 µg), and trimethoprim (5 µg). *Escherichia coli* ATCC 25922 and *K. pneumoniae* ATCC 700603 were used in each batch as quality controls. Meropenem Etest (Biomérieux) was used for minimum inhibitory concentration testing, which was carried out on isolates selected for plasmid conjugation assays and their transconjugants. Results were interpreted using EUCAST clinical breakpoints (v 12.0) except for streptomycin, tetracycline, and kanamycin which were interpreted using Clinical and Laboratory Standards Institute (CLSI) guidelines (M100:ED32). Differences in the total number of instances of phenotypic resistance displayed between environmental and human isolates, and between OXA-48-, KPC-, and NDM-producing isolates, were assessed by Fisher’s exact test, with significance set at *P* = 0.05 by convention. Two-by-two contingency tables were generated for each comparison with the sum of observed phenotypic resistances and susceptibilities (defined as susceptible or intermediate) compared across all antimicrobials for each isolate category. To account for correlated resistance patterns between clonally related isolates, only one randomly selected representative of each genetically related cluster [<21 single nucleotide polymorphisms (SNPs) distance] was included in the analysis.

### Whole-genome sequencing and *de novo* assembly

All isolates were sequenced using both NGS and third-generation sequencing methods to obtain short- and long-sequencing reads, respectively. For short-read sequencing, DNA was extracted from all isolates using the EZ1 advanced XL machine and the EZ1 DNA tissue kit (Qiagen). Isolates from the NCPERL were sequenced using the Illumina MiSeq and the Illumina DNA Flex library preparation kit (PE300). Environmental isolates were short-read sequenced as described previously, using the Illumina NovaSeq 6000 in Oxford’s Genomics Centre (PE150) (17, 18). For long-read sequencing, DNA was extracted using the QiaAmp DNA mini kit (Qiagen), using a low-shear protocol. DNA quality and concentration were assessed using the DeNovix DS-11 spectrophotometer/fluorometer and Qubit (Invitrogen Corp., Carlsbad, CA, USA) platforms. Sequencing was carried out using the MinION platform (Oxford Nanopore Technologies Ltd., Oxford Science Park, Oxford, United Kingdom). Libraries were prepared using the EXP-CTL001and SQK-RBK004 kits (Oxford Nanopore Technologies, Ltd.) and run on FLO-MIN106 flow cells for at least 48 h. Basecalling of raw Fast5 files was via Guppy, a neural network-based basecaller using the default settings (MinKNOW v22.12.5). The average short-read depth achieved was 133 (±90), and the average long-read depth was 52 (±45).

Quality control and adapter trimming of short-read sequences were carried out using fastp (v0.23.2) (22). Reads with a Phred quality (Q) score of less than Q15 and length of less than 15 bp were filtered out using default parameters. Sequence quality control and filtering of long reads were carried out using filtlong (v0.2.1; Wick, R. R. (2018a) Filtlong. San Francisco, CA: GitHub] using default settings, with extensions --min_length 1,000 and –keep_percent 95. Hybrid genome assembly was carried out on all samples using Unicycler (v0.4.8) (23) with default parameters. The quality of hybrid assemblies was assessed using QUAST (v5.2.0; see Table S5).

### Genotypic characterization of CPE isolates

MLST (v2.23.0) was used to check the species identification and to determine the MLST of all isolates. The appropriate PubMLST scheme was automatically selected based on the species designation (Seemann, T., https://github.com/tseemann/mlst).

Genome assemblies were visualized using Bandage (v0.81) (24). Closed genomes were obtained for 25/39 isolates. Chromosomal and plasmid assemblies were sorted into separate FASTA files, and only closed, circular plasmids were used in downstream analysis. All files were annotated using Bakta (v1.4.0) to determine the complete gene content and gene families. Annotation was via a large taxonomy-independent database (downloaded 8 April 2022) using UniProt’s entire UniRef protein sequence cluster universe (25).

Genome assemblies were analyzed with ABRicate (v1.0.1; Seemann T, 2019 Abricate, Github https://github.com/tseemann/abricate) using the ResFinder (26) and PlasmidFinder (27) databases to determine the resistance genes and plasmid content, respectively. Both were run using default settings of 80% identity and coverage.

Snippy (v4.6.0; https://github.com/tseemann/snippy) was used to compare genomes that had the same MLST. Genomes were compared to a reference genome of the same MLST. The reference genomes per ST for this study were randomly selected from the genomic options available per ST. The assembly statistics of the reference genomes can be found in Table S6. A SNP threshold of 21 has been used to discriminate clonal isolates as previously described by David et al. (28).

An all-vs-all average nucleotide identity (ANI) analysis using ANIcluster map (v1.2.0; https://github.com/moshi4/ANIclustermap), which is based on the fastANI and seaborn algorithms, was used to search for closely related plasmids. BLAST was used to determine the percentage coverage and percentage identity of the plasmids. Plasmids carrying the same CEG which clustered closely (>98% ANI) and had high identity (>98%) and coverage (>80%) were aligned and visualized using Blast Ring Image Generator (BRIG, v0.95) (29).

TETyper (v1.1) was used to analyze the environment around the transposable elements within plasmids for the presence of repeated motifs. The Tn*4401b* reference provided profile definitions for single nucleotide variants/deletions, which were used to determine the transposon variant and the flanking regions of the *bla*_KPC_ genes (30).

Visualization of data was achieved using RStudio (2022.07.0+548), BRIG (29), and Clinker (v0.0.23) (31).

### Conjugation study

The ability of selected study isolates to transfer their carbapenemase plasmid by conjugation was assessed by filter mating as described by Kang et al. (32), with modifications. Plasmids with an undetermined ability to conjugate, due to size or loss of genes, were chosen. Sodium azide-resistant *E. coli* J53 was used as the recipient. Transconjugants were selected on Luria Bertani Agar (LBA) containing cefotaxime (1 mg/mL) plus sodium azide (100 µg/mL), and recipients were selected on LBA with sodium azide alone. Serial dilutions of mating mixtures were plated in triplicate, and the conjugation frequency was calculated as the mean number of transconjugants divided by the mean number of recipients [adapted from reference (32)].

## RESULTS

### Antimicrobial-resistant Enterobacterales species, phenotypes, and genotypes

The collection of 39 CPE isolates comprised four different genera and eight distinct species, with the most prevalent species *K. pneumoniae* (*n* = 14) and *E. coli* (*n* = 13), as detailed in Table S1. Among the *Klebsiella* species*, K. oxytoca* (*n* = 1)*, K. michiganensis* (*n* = 1)*,* and *K. pasteurii* (*n* = 3) were exclusively identified in patient samples, while *K. variicola* (*n* = 1) was solely detected in wastewater samples. All isolates except for one (KO190245) exhibited multidrug-resistant phenotypes with a median of resistance to six antimicrobial classes (Table S1).

Isolates producing NDM displayed a significantly greater proportion of antimicrobial-resistant phenotypes than those producing OXA-48 (Fisher exact test *P* = 0.0014). CPE of environmental origin displayed a greater proportion of phenotypic resistance than those of human origin; however, this finding was not significant (Fisher exact test *P* = 0.1211).

### Sequence types and analysis of clonal dissemination

#### *Sequence types associated with bla*_*OXA-48*_ *carriage*

**Fig 1:**
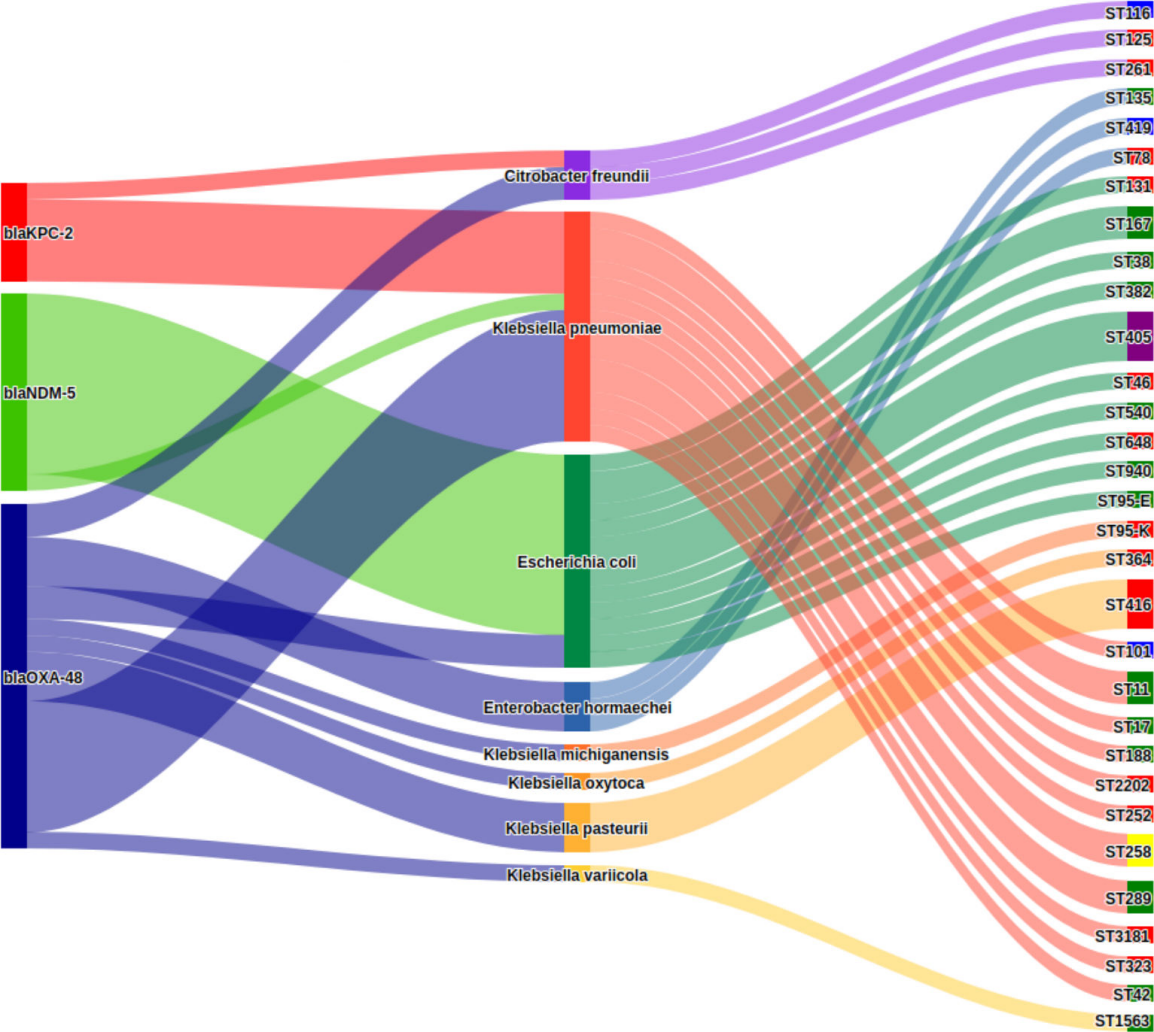
Sankey diagram representing the species and sequence types (STs) associated with each carbapenemase gene. STs are color-coded depending on the source: green = natural environment; red = human; blue = hospital environment; purple = both human and natural environment; and yellow = both human and hospital environment (see Table S1 for more detail).

The collection of 21 *bla*_OXA-48_ carrying isolates spanned eight different species and 18 different STs. The dominant genus was *Klebsiella*, with 15 isolates. Three significant clusters within the *Klebsiella* isolates were identified and classified by their sequence types ST11, ST289, and ST416 (Fig. 1). These clusters were remarkably similar genetically, with an ANI greater than 99.99% and less than 10 SNPs separating them, indicating a very high degree of genetic similarity (Table 1). Each cluster was consistently found in the same type of environment, either in humans or in environmental samples.

The first cluster consisted of two ST11 *K. pneumoniae* environmental isolates which were previously reported by Hooban et al. (17, 18). The first of these isolates was identified in an estuary sample (B18235), and 2 days later, the second isolate (B18291) was retrieved from a seawater sample from a nearby beach, under 3 km away (18).

The second cluster of *K. pneumoniae* ST289 (*n* = 2) was identified in two seawater samples collected on the same day from beaches within 2 km of each other (17)

The final cluster consisted of *K. pasteurii* ST416 (*n* = 3) originating from rectal swabs of three distinct patients from the same hospital collected within 6 months.

#### *Sequence types associated with bla*_*KPC-2*_ *carriage*

The KPC-producing isolates consisted of *K. pneumoniae* (*n* = 5) and *Citrobacter freundii* (*n* = 1) (Fig. 1). The *bla*_KPC-2_ gene was detected on a plasmid in all but one environmental (seawater) isolate of *K. pneumoniae* (B19293).

Two isolates were *K. pneumoniae* ST258, which is an epidemic high-risk clone associated with the global dissemination of *bla*_KPC_ (33). These ST258 isolates, sharing >99.8% ANI and differing by 48 SNPs, were initially identified in hospital sewage and 5 months later in a patient’s rectal swab. Despite their high ANI, the ST258 isolates presented distinct plasmid and resistance gene profiles (Table 1; Table S1).

#### *Sequence types associated with bla*_*NDM-5*_ *carriage*

The NDM-producing isolates consisted of *E. coli* (*n* = 11) and *K. pneumoniae* (*n* = 1). The *E. coli* isolates exhibited eight different STs, including ST405 (*n* = 3), ST167 (*n* = 2), ST131 (*n* = 1), and ST648 (*n* = 1) which are high-risk clones commonly associated with *bla*_NDM_ carriage (34, 35) (Figure 1) .

All environmental isolates harboring *bla_NDM-5_* were typed as *E. coli*, with ST405 (*n* = 2) and ST167 (*n* = 2) comprising 4/7 of the environmental isolates. Two ST167 (B20127 and B20159) isolates, collected from adjacent beaches within 18 days, shared >99.8% ANI and differed by 145 SNPs. Both isolates shared almost identical *bla*_NDM-5_ harboring plasmids (Table 2; Table S2); however, B20159 carried an additional resistance plasmid carrying *aph(6)-Id* and *sul2*.

Three *E. coli* ST405 isolates (B19429, B20105, and EC190329) displayed similar but distinct resistance gene content and phenotypes and carried *bla*_NDM_ plasmids with different combinations of IncF-type replicons. Interestingly, the two environmental *E. coli* ST405, isolated within 6 months of each other in two locations on the same estuary, were within seven SNPs of each other and carried 98% similar carbapenemase plasmids, but had differing AMR and plasmid profiles (Table 1) .

**TABLE 1:** Spatiotemporal and clonality comparison of CPE sequence types

ST	Species	CEG	Isolate ID	Sample type	Date of collection	Distance (km)	No. of SNPs^*a*^
11	*K. pneumoniae*	*bla*_OXA-48_	B18235	Estuary	December 2018	2.8	**5,488**
			B18291	Seawater	December 2018		**5,479**
289	*K. pneumoniae*	*bla*_OXA-48_	B20154	Seawater	March 2020	1.8	**26,502**
			B20158	Seawater	March 2020		**26,509**
416	*K. pasteurii*	*bla*_OXA-48_	KO190356	Human rectal	July 2019	0	**22,253**
			KO190322	Human rectal	August 2019		**22,261**
			KO200862	Human rectal	December 2020		**22,261**
258	*K. pneumoniae*	*bla*_KPC-2_	B18188	Hospital sewage	November 2018	0.1	9,412
			KP1902196	Human rectal	April 2019		9,364
405	*E. coli*	*bla*_NDM-5_	EC190329	Human rectal	February 2019	1.5	5,141
			B19429	Estuary	August 2019		**3,210**
			B20105	Estuary	February 2020		**3,203**
167	*E. coli*	*bla*_NDM-5_	B20127	Seawater	February 2020	0.84	2,369
			B20159	Seawater	March 2020		2,224

^*a*^ Number of SNPs from the reference genome: GCF_947391185.1 (ST11), SRR3051116 (ST289), SRR11046432 (ST416), SRR16761272 (ST258), GCF_022699465.1 (ST167), and GCF_947390085.1 (ST405). Isolate pairs considered clonally related are in bold, informed by SNP distance, AMR phenotype/genotype, and plasmid content.

### Analysis of carbapenemase-bearing plasmids

#### *Analysis of IncL bla*_*OXA-48*_ *plasmids*

The predominant plasmid replicon type associated with *bla*_OXA-48_ was IncL, detected in 18/21 (86%) OXA-48-producing isolates. Plasmid sizes varied from 48 to 86 kb. A highly similar (>99.9% ANI) 63.6 kb IncL *bla*_OXA-48_ plasmid was detected in 13/21 (62%) OXA-48-producing isolates. This conserved broad host range plasmid was detected in 11 different STs across five different bacterial species and in isolates from both human and environmental domains. Five isolates from human samples [blood (*n* = 2) and rectal swabs (*n* = 3)] carried this plasmid, and it was detected in isolates from three different sub-domains of environmental samples [seawater (*n* = 5), river estuary (*n* = 2), and hospital (*n* = 1)]. It transferred to *E. coli* J53 from *K. pneumoniae*, *K. pasteurii*, and *Enterobacter hormaechei* with conjugation efficiencies of 0.67%, 3.2%, and 98.4%, respectively (Table S4).

The remaining five IncL plasmids identified had high sequence homology (>98.5% ANI) but varied in terms of regulatory and conjugation gene content. Specifically, a *K. oxytoca* (KO190245) and a *K. pasteurii* (KO190356) carried IncL plasmids which were significantly smaller, approximately 48 kb. Neither of these plasmids had an origin of replication, and they lacked the relaxase genes associated with plasmid mobility. KO190356 is highly similar clonally (>99.99% ANI) to another isolate KO200862 which also carried a truncated IncL plasmid (~54 kb) but retains the machinery necessary for self-mobilization. All three of these isolates also carry an additional ~48 kb conjugative IncX3 plasmid bearing a *qnrS1* gene. All IncL plasmids we assessed that lacked the necessary machinery failed to conjugate at a detectable rate. Moreover, no co-transfer of the truncated IncL plasmid with the IncX3 plasmid was recorded during the plasmid conjugation study.

#### *Analysis of IncM1 bla*_*OXA-48*_ *plasmids*

**Fig 2:**
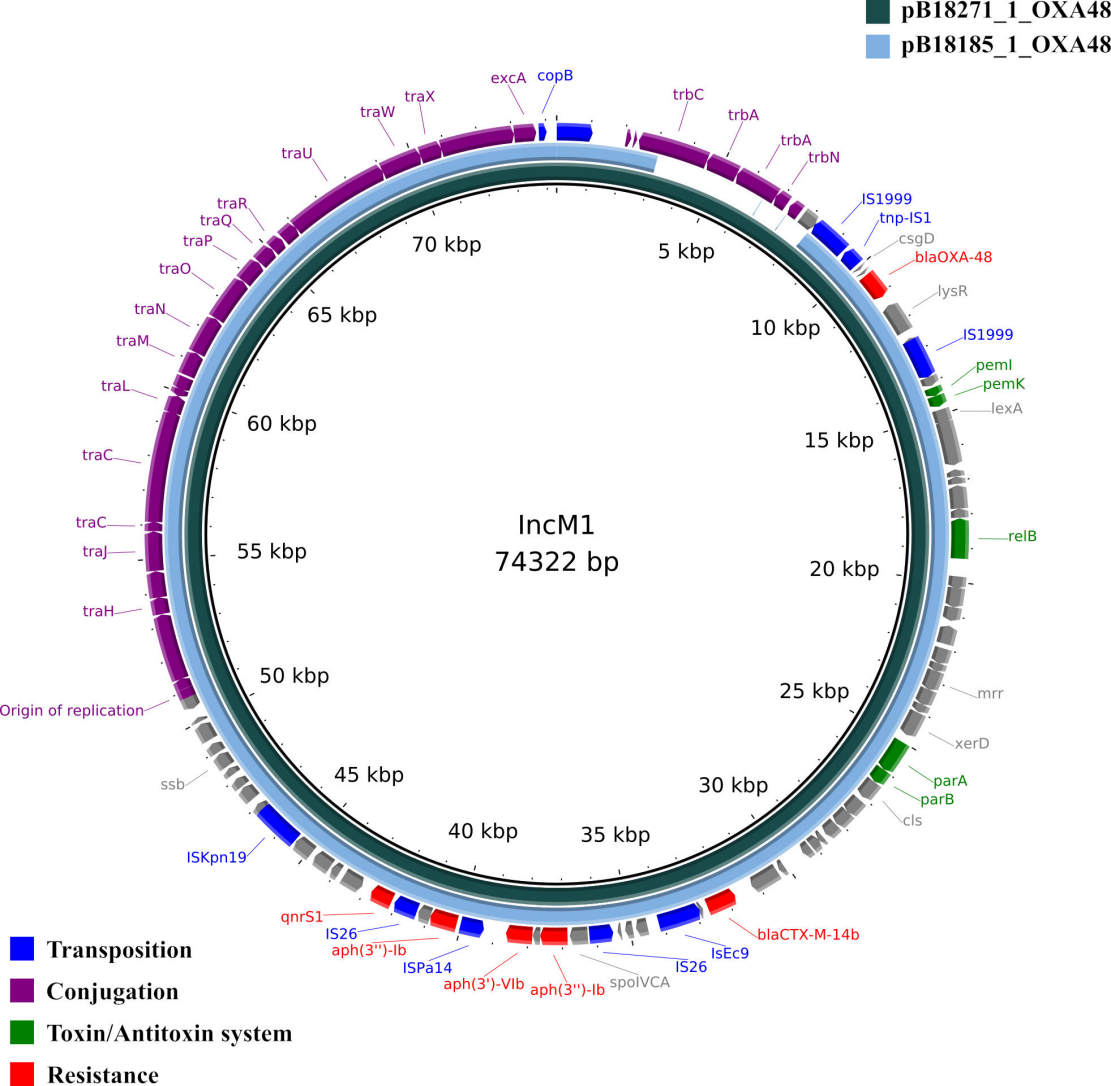
Comparison of IncM1 plasmids. The inner rings represent the genome of each plasmid pB18271_1_OXA48 = *E. coli* ST540 and pB18185_1_OXA48 = *K. pneumoniae* ST101 (93% coverage), according to the legend in the top right corner. The outer ring represents the different genes detected and colored according to the legend in the bottom left corner.

In two isolates, the *bla*_OXA-48_ gene was encoded on an IncM1 plasmid of the MOBP relaxase family and type I mating pair formation (MPF) system (Fig. 2). The IncM1 plasmids were detected in *E. coli* ST540 (pB18271_1_OXA48) and *K. pneumoniae* ST101 (pB18185_1_OXA48) isolated from seawater and hospital sewage, respectively, within a 9-day period. The plasmids were highly similar (99.85% ANI) in both isolates; however, in pB18185_1_OXA48, it lacked an MPF and the *trbA-C* genes which are essential for conjugation (36). This was borne out in the conjugation study results, where the 74 kb IncM plasmid in B18271 conjugated with an efficiency of 5.5%, but the 70 kb plasmid pB18185_1_OXA48 was not conjugative at an appreciable efficiency (Table S4).

#### *Analysis of bla*_*KPC-2*_ *plasmids*

The *bla*_KPC-2_ genes were predominantly associated with IncF mutlireplicon plasmids except for one chromosomally encoded *bla*_KPC-2_ in a *K. pneumoniae* ST188 seawater isolate. All IncF plasmids in *K. pneumoniae* contained MOBF family relaxases and type F MPF. However, in a *C. freundii* ST116 isolate from hospital sewage (pB18196_1_KPC2), the *bla*_KPC-2_ gene was carried on the only plasmid in the collection with multiple different types of replicons, IncF (FII/FIB) and IncX3. This 198 kb plasmid had both MOBP and MOBF family relaxases and type F MPF systems.

Four IncF plasmids in *K. pneumoniae* ST258 (pKP1902196_1_KPC2:318Kb and pB18188_1_KPC2:117Kb), *K. pneumoniae* ST42 (pKP2009864_1_KPC2:301Kb), and ST2202 (pKP1806281_1_KPC2: 128Kb) shared common replicon types and resistance genes (Table S2).

**Fig 3:**
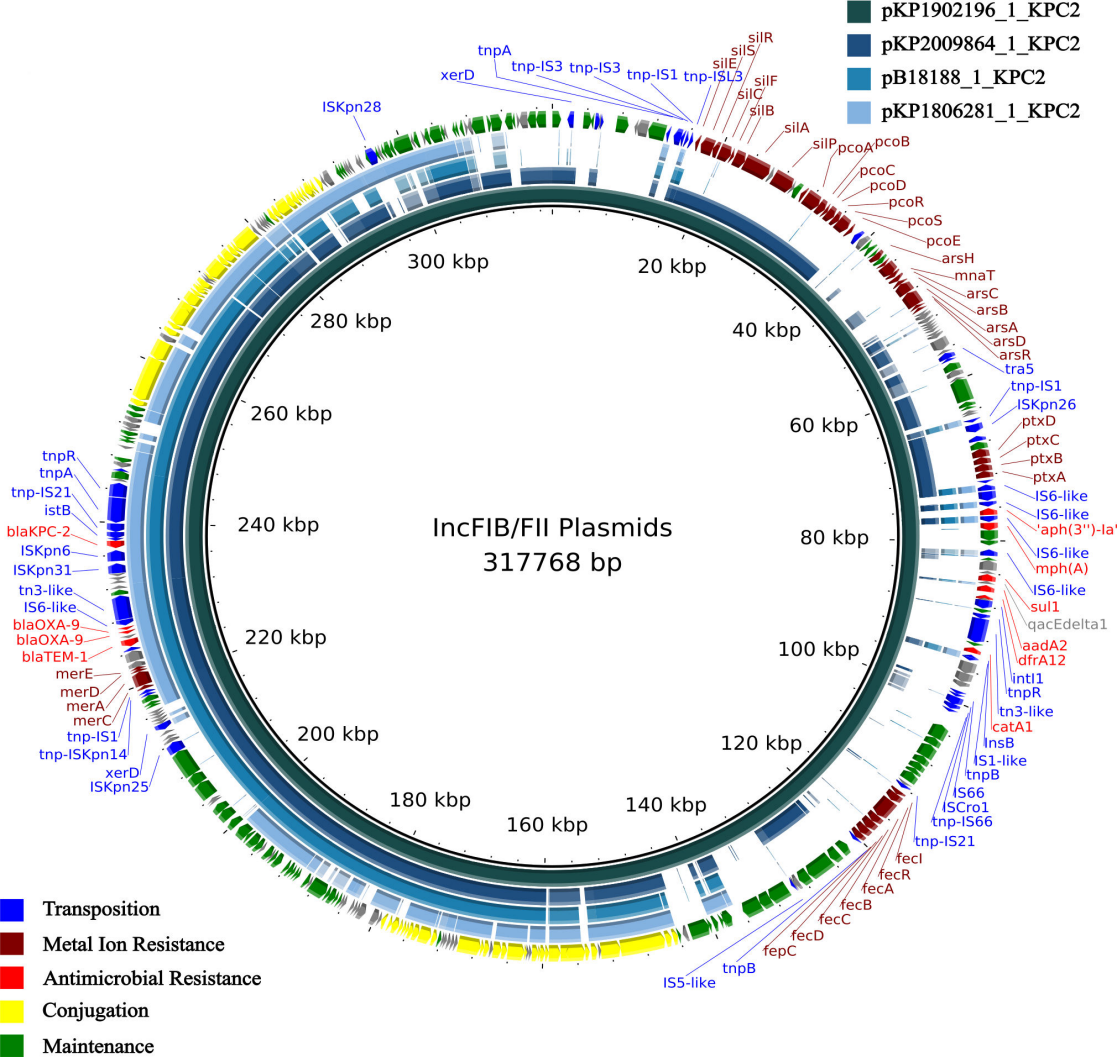
Comparison of bla_KPC-2_ bearing plasmids. A 318 kb plasmid detected in a human rectal swab isolate (pKP1902196_1_KPC2) displayed as the inner ring. The next three rings represent similar plasmids detected from a rectal swab (pKP2009864_1_KPC2, 70% coverage), hospital sewage (pB18188_1_KPC2, 49% coverage), and human stool (pKP1806281_1_KPC2, 43% coverage). The blue rings represent the genome of each isolate according to the legend in the top right corner. The outer ring represents the different genes detected and colored according to the legend in the bottom left corner.

These plasmids shared at least 99.2% nucleotide identity to a portion of the larger (318 kb) plasmid in a *K. pneumoniae* ST258 (pKP1902196_1_KPC2) isolated from a rectal swab in 2019. The plasmids displayed variable carriage of insertion sequence bound metal ion resistance regions, with resistance genes to mercury present in all, silver, copper, and arsenic in the two larger plasmids, and iron found only in the largest plasmid (Fig. 3). The larger plasmid also carried a class 1 integron with additional resistance genes to aminoglycosides [*aadA2, aph(3”)-Ia*], trimethoprim (*dfrA12*), sulfonamides (*sul1*), azithromycin (*mphA*), and chloramphenicol (*catA1*). Despite carrying all the genes necessary for conjugation, attempts to conjugate this plasmid into *E. coli* J53 were unsuccessful (Table S4).

#### *Analysis of bla*_*NDM-5*_ *plasmids*

The *bla*_NDM-5_ gene was carried on multireplicon IncF plasmids in 11/12 isolates, which ranged in size from 115 to 148 kb and carried a median of six antimicrobial resistance genes (ARGs; ranges 4–8). These IncF plasmids were clustered into four groups based on ANI, resistance gene profiles, and percentage coverage and identity, with each group mainly found in similar sources.. Two plasmid pairs within these groups had identical IncF replicon types, suggesting a shared plasmid structure. Notably, two plasmids of type IncFIA/FIC(FII) were found in the same ST (ST167) isolates, which were not deemed closely related.

There was evidence of potential dissemination of *bla*_NDM-5_ encoding plasmids with identical IncF type across different STs, ecological niches, and species. IncFIA/FII plasmids were detected in a human *E. coli* ST46 isolate and a river *E. coli* ST405 isolate. Of note, the human isolate’s plasmid also encoded pan-aminoglycoside resistance gene *rmtB*. IncFIB/FII plasmids were discovered in *K. pneumoniae* ST3181 (pKP1906522_1_NDM5) and *E. coli* ST131 (pEC190759_1_NDM5) from different patients in the same hospital, identified within 2 months. Both plasmids contained *bla*_NDM-5_ within a distinct IS*26*-bound transposon, alongside *aph(3)-VI* and *qnrS1* genes. These plasmids also featured integrons with *sul1*, though their additional resistance gene arrays varied (Fig. 6).

The sole non-IncF *bla*_NDM-5_ plasmid was detected in an *E. coli* ST940 (pB19522_1_NDM5) seawater isolate which carried the globally dominant 46 kb IncX3 plasmid, recently designated pX3_NDM-5 (37). This plasmid shared >99.9% sequence identity to similar plasmids from China (CP060888.1), Bangladesh (CP095642.1), and Japan (AP023210.1). Additionally, this isolate contained an IncFIA/FIB/FIC(FII) plasmid with 98.8% identity and 72% coverage of plasmids with *bla_NDM-5_* in *E. coli* ST405, ST167, and ST46 from river estuary, seawater, and human samples. Both isolates were collected from aquatic environments close to each other within a month (Table 2).

**TABLE 2:** Summary of bla_NDM_-bearing plasmids

Group	ID	Species	Sequence type	Incompatibility group	Additional resistance genes^*a*^	Size (bp)	Source	Identity	Coverage
1	pEC190486_1_NDM5	*E. coli*	46	IncFIA/FII	*aadA2, dfrA12, mphA, sul1, tetA, bla*_TEM-1B_, ***rmtB***	124,286	Human	100.00%	100%
	pB20105_1_NDM5	*E. coli*	405	IncFIA/FII	*aadA2, dfrA12, mphA, sul1, tetA*	118,996	Estuary	99.97%	95%
	pB20159_1_NDM5	*E. coli*	167	IncFIA/FIC(FII)	*aadA2, dfrA12, mphA, sul1, tetA*	120,444	Seawater	99.98%	92%
	pB20127_1_NDM5	*E. coli*	167	IncFIA/FIC(FII)	*aadA2, dfrA12, mphA, sul1, tetA*	120,441	Seawater	99.98%	92%
	pB19429_1_NDM5	*E. coli*	405	IncFIA/FIB/FIC(FII)	*aadA2, dfrA12, mphA, sul1, tetA,* ***bla***_**TEM-1B**_	120,124	Estuary	98.30%	79%
2	pEC190759_1_NDM5	*E. coli*	131	IncFIB/FII	*aph(3')-VI, qnrS1, sul1,* ***aadA5, drfA17***	137,130	Human	100.00%	100%
	pKP1906522_1_NDM5	*K. pneumoniae*	3181	IncFIB/FII	*aph(3')-VI, qnrS1, sul1,* ***aac(6')-II, bla***_**OXA-10**_	134,096	Human	99.98%	96%
3	pB20123_1_NDM5	*E. coli*	95	IncFIA/FIB/FII	*aadA2, dfrA12, sul1*	148,373	Seawater	100.00%	100%
	pB19374_1_NDM5	*E. coli*	382	IncFIA/FIB/FII	*aadA2, dfrA12, sul1*	142,988	Seawater	99.70%	86%
4	pEC190329_1_NDM5	*E. coli*	405	IncFIB/FII	***aadA2****, bla*_TEM-1B_, ***dfrA12, mphA, qepA4,*** *sul1, tetB*	129,346	Human	100.00%	100%
	pEC200461_1_NDM5	*E. coli*	648	IncFIB/FIC(FII)	***aadA5****, bla*_TEM-1B_, ***dfrA17****, sul1, tetB*	115,135	Human	100.00%	86%
N/A^*b*^	pB19522_1_NDM5	*E. coli*	940	IncX3	None detected	46,161	Seawater	None detected	None detected

^*a*^ Genes highlighted in bold differ between similar plasmids.

^*b*^ N/A, not applicable.

### Genetic environment surrounding CEGs

#### *Tn1999 transposon containing bla*_*OXA-48*_

**Fig 4:**
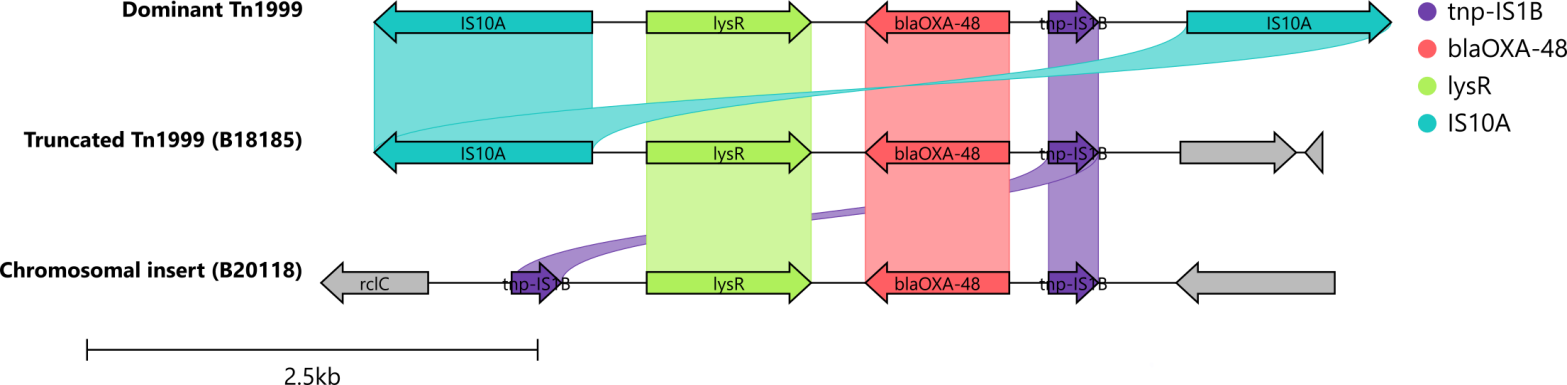
Comparison of genetic environment surrounding bla_OXA-48_ genes. The dominant Tn1999 (top) was found in 19/20 bla_OXA-48_ plasmids, while a single IncM plasmid carried a truncated version. In a single isolate, bla_OXA-48_ was located on the chromosome and only carried some of the Tn1999 elements.

In 19 out of 20 *bla*_OXA-48_ containing plasmids, a 10 kb Tn*1999* transposon was identified, closely resembling the Tn*1999.2* variant. This Tn*1999* transposon was found in both IncL and IncM1 plasmids, featuring a 13 bp terminal inverted repeat (IR) and a 9 bp target site duplication (TSD). However, in one IncM1 plasmid from *K. pneumoniae* ST101, this region was incomplete. In one case, an *E. coli* ST38 isolate had the *bla*_OXA-48_ gene integrated into its chromosome, accompanied by the *lysR* gene from Tn*1999* but missing other transposon elements, and instead was bordered by two IS*1B* sequences in direct orientation with a 15 bp direct repeat near the IS*1B* (Fig. 4).

#### *Tn4401 transposon containing bla*_*KPC-2*_ *gene*

**Fig 5:**
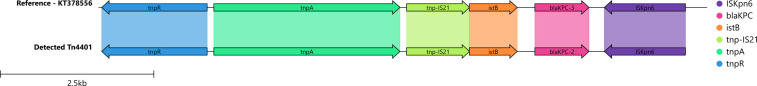
The Tn4401a transposon detected in all bla_KPC-2_ bearing isolates. The region was identical in all genomes and was a match for the Tn4401a transposon.

An identical transposon with 100% identity to Tn*4401a* was found in all *bla*_KPC-2_ bearing isolates, both those with the gene located on plasmids and in the isolate where it was encoded on the chromosome. All transposons were surrounded by an imperfect 40 bp terminal IR and a 5 bp TSD. The IR was the same in all isolates, but the TSD differed in isolates where *bla*_KPC-2_ was plasmid (ATTGA|ATTGA) and chromosomally (AATAT|AATAT) encoded (Fig. 5).

#### *IS26 bound region surrounding bla*_*NDM-5*_ *gene*

**Fig 6:**
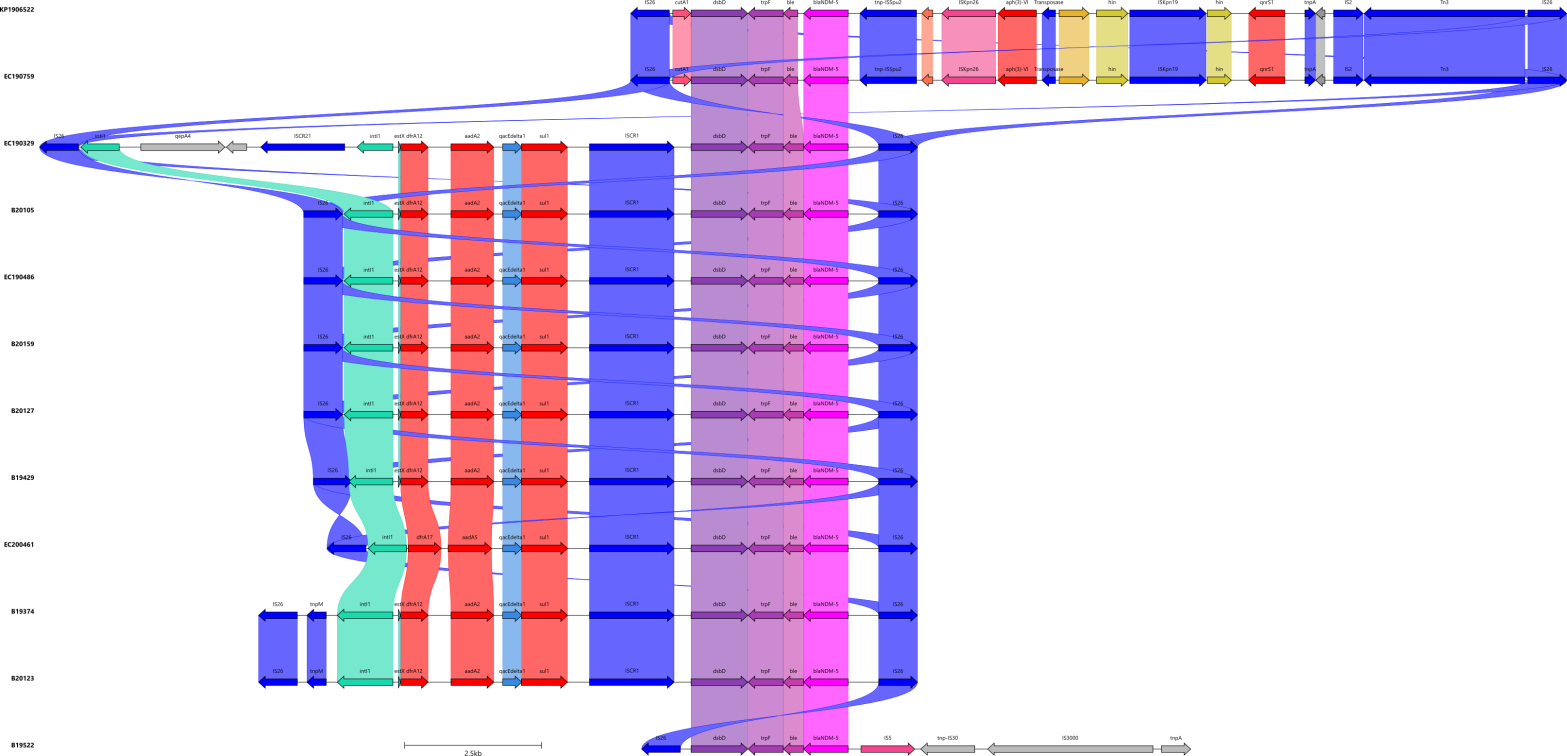
Genetic environment surrounding the *bla*_NDM-5_ gene in each plasmid. The *bla*_NDM-5_ gene is in pink, while other resistance genes are in red. Insertion sequences are highlighted in blue.

The region surrounding the *bla*_NDM-5_ gene was highly conserved in all plasmids, containing the *bla*_NDM-5_, *ble*_MBL_, *trpF*, and *dsbD* genes. In 9/12 plasmids (75%), these genes were within a composite transposon bound by IS*26* which also contained an IS*CR1* element and a class 1 integron with the *dfrA12-aadA2-sul1* gene cassette. Two isolates that shared IncFIB/FII plasmids (pEC190759_1_NDM and pKP1906522_1_NDM) had a very different composite transposon from the others that included Tn*3* and resistance genes *aph(3)-VI* and *qnrS1*. No additional resistance genes were detected in the IcX3 plasmid, where the conserved *bla*_NDM-5_ structure was located between IS*26* and IS*5* (Fig. 6).

### Additional resistance plasmids

In 95% (37/39) of isolates sequenced, at least one extra plasmid was detected, up to a maximum of seven additional plasmids in some isolates (mean plasmid number = 3.7; range = 1–8). An additional resistance plasmid was carried by 62% (24/39) of isolates, while a single isolate (B20159) carried two additional resistance plasmids. These plasmids were most commonly IncF-type plasmids, followed by IncX-type plasmids (Table S3).

Two IncX3 plasmids in *K. pneumoniae* ST258 (pKP1902196_2) and *K. pasteurii* (pKO200862_3) harbored *bla*_SHV-182_ and *qnrS1*, respectively. These plasmids shared high identity (99.99% and 92.95%) and coverage (85% and 72%) with the IncX3 plasmid carrying *bla*_NDM-5_ in B19522 (pB19522_1_NDM5). All three plasmids shared a common backbone but contained potentially transposable regions carrying the variable resistance genes.

Of note, an *E. hormaechei* isolate, EN200458, harbored a 293 kb resistance plasmid (pEN200458_2_VIM) carrying the *bla*_VIM-1_ and *mcr-9* genes in addition to the IncL *bla*_OXA-48_ plasmid. This IncH plasmid was MOB type H but lacked any MPF genes, suggesting it may be mobilizable but not self-conjugative. This was confirmed in the conjugation study where the IncL plasmid carrying *bla*_OXA-48_ was transferred at a very high rate (98%), but the IncH type plasmid carrying *bla*_VIM-1_ failed to transfer at a detectable rate (Table S4).

## DISCUSSION

This study analyzed the MGE associated with dissemination of *bla*_OXA-48_, *bla*_KPC-2_, and *bla*_NDM-5_ genes among human and environmental Enterobacterales in Galway, Ireland. The results demonstrated variable complexity based on the specific carbapenemase gene. All three CEGs were linked to specific transposable elements on plasmids and, for *bla*_OXA-48_ and *bla*_KPC-2_, also on bacterial chromosomes.

The research findings further highlighted that the transfer of the CEG and MGE across the genomic boundaries of different strains, species, and genera may be a more important factor in the spread of *bla*_KPC-2_ and *bla*_NDM-5_ than clonal expansion in this location. Identical plasmids encoding for *bla*_OXA-48_ and nearly identical plasmids carrying *bla*_KPC-2_ were found across different strains and species in both human and environmental samples. These findings are in accordance with environmental identification of CPE in Ireland reported by Hooban et al. (17, 18). The results highlighted that the spread of certain *Klebsiella* sp. clones partly explained the local transmission of *bla*_OXA-48_. Although high-risk CPE clones linked to human infections were found in the natural environment, no common clonal groups were observed in multiple compartments in this study.

This study supports the connection of *bla*_OXA-48_ with the IncL plasmid known for its wide host range and ability to transfer between Enterobacterales species in this region. Interestingly, in two cases, *bla*_OXA-48_ was relocated to a larger IncM1 plasmid with more antimicrobial resistance genes, identified in hospital sewage. The reference laboratory has not detected IncM1 in isolates from human sources; however, methods, which rely on short-read sequences, cannot reliably distinguish this IncM1 plasmid from the common IncL plasmid. This may lead to errors in mapping chains of plasmid transmission in suspected outbreaks. The findings also suggest that the IncM1 plasmid’s transfer efficiency might be lower than that of IncL, possibly varying by species.

The detection of *bla*_KPC-2_, primarily within the MGE Tn*4401*, with dissemination across various IncF type plasmids and chromosomally, indicates a high-frequency *bla*_KPC_ mobilization (38). *bla*_KPC-2_ was almost exclusively associated with *K. pneumoniae*, except for a *C. freundii* isolated from hospital sewage, which bore the only CEG plasmid in the collection with both IncF and IncX3 replicons. Such plasmids are often produced via the fusion of single replicon plasmids, which enhances ARG diversity and host range (39). We did not find direct evidence of fusion of resistance plasmids in related isolates. Although direct evidence of plasmid fusion was absent, 62% of isolates had additional AMR plasmids, some similar to CEG plasmids from environmental isolates, like the IncF plasmid in an *E. coli* isolate from seawater.

Comparison of all *bla*_KPC_ plasmids collected from 2018 to 2020 in Galway allowed us to trace possible local evolution dynamics of these resistance plasmids over this period. A group of closely related plasmids included the 317 kb (pKP1902196_1_KPC2) plasmid, with metal ion and antimicrobial resistance genes located within MGE, and its three analogs which displayed variable carriage of these MGE (Fig. 3). The patient isolate collected in April 2019 had the most resistance genes, whereas the other plasmids only carried the same three resistance genes. This highlights the mosaic nature of these multireplicon plasmids and how frequent recombination events involving MGEs can lead to many diverse plasmid structures carrying a wide range of resistance genes (8). Metal ions, such as silver (40) and copper (41, 42), are commonly used in antibacterial dressings, medical devices, and disinfectants, which may explain the detection of these genes in isolates from patients and clinical environments. The detection of such genes on MGE-bearing *bla*_KPC_ genes (co-resistance) is of concern, considering the propensity for co-selection of *bla*_KPC_ plasmids through the use of healthcare disinfectants (43). Moreover, two such isolates were *K. pneumoniae* ST258, an epidemic clonal lineage with outbreak potential that has contributed greatly to the dissemination of *bla*_KPC_ (33). However, there is a cost associated to the cell with maintaining a large plasmid, and as suggested by the conjugation study results, their ability to transfer may be diminished. Accordingly, the ability to gain and lose MGE-bound resistance modules to suit the environment, as may have occurred here, represents a mechanism that may be associated with a competitive advantage for plasmid success.

The MGE responsible for mobilizing *bla*_NDM-5_ was more complex, involving IS*26*-bound composite transposons with a conserved core structure around *bla*_NDM-5_, (*bla*_NDM-5_*-ble*_MBL_*-trpT-dsbD*). Each possessed at least one additional insertion sequence or transposase and several ARG and were plasmid-borne. IS*26* has demonstrated a great capacity for structural reorganization of resistance plasmids through replicative transposition (44).

All but one *bla*_NDM-5_ plasmid was of IncF type, and most carried multiple toxin-antitoxin genes, which function to ensure plasmid maintenance in daughter cells and can potentially contribute to persistence and/or virulence (45). They were detected in a wide array of nine *E. coli* STs, and in a human *K. pneumoniae* ST3181 isolate, the plasmid of which bore striking resemblance to that of another human isolate, *E. coli* ST131, collected 2 months later. Generally, *bla*_NDM-5_ plasmids that were more similar to each other were found in bacteria isolated from the same ecological niche (Table 2; Fig. S3). However, one example of a highly similar *bla*_NDM-5_ plasmid (IncFIA/FII) in both human (pEC190486_1_NDM5) and environmental (pB20105_1_NDM5) isolates was found. Chronologically, the plasmid was first detected in a human *E. coli* ST46 isolate in 2019, followed by detection in ST405 in a river sample the following year. This could indicate contamination of the environment by effluent of human origin originating from the hospital or from human sources living in the community. This hypothesis is further supported by the detection of high-risk extraintestinal pathogenic *E. coli* (ExPEC) clones ST167 and ST405 in the environment, which were recently identified as the two most common STs carrying *bla*_NDM-5_ in clinical isolates from 13 European countries (46). The plasmids differ in their ARG content, with both *rmtB* and *bla*_TEM-1B_ present in ST46 but lacking in the river ST405 isolate plasmid collected in 2020. Turton et al. (47) have reported a high rate of co-carriage of *rmtB* and *bla*_NDM-5_ in *E. coli* patient isolates from England since 2013, and hybrid WGS analysis has revealed their co-carriage on multireplicon IncF plasmids as described herein. IncF *bla*_NDM-5_ plasmids with co-carriage of *rmtB* have been described in several continents, including South America (43), Asia (48–50), and in other European countries (46, 51, 52). If *bla*_NDM-5_ plasmid transmission between STs occurred, it is more likely to have occurred in environments with high concentrations of donor and host, such as in the gastrointestinal tract or effluent likely to contain high numbers of potential donors such as hospital sewage.

The only non-IncF *bla*_NDM-5_ plasmid was detected in a seawater ST940 *E. coli* isolate, which contained the globally dominant 46 kb IncX3 plasmid. Dissemination of *bla*_NDM-5_ on IncX3 plasmids has been associated with multispecies hospital outbreaks (53, 54) and has been reported worldwide including China (55), Korea (56), Japan (57), Mali (58), USA (59), and the United Kingdom (60). The IncX3 NDM-5 plasmid was recently shown to be capable of interphylum dissemination, even capable of transferring from Gram-negative to Gram-positive species, and back again, under simulated hospital wastewater treatment plant conditions (37). Interestingly, we detected similar-sized IncX3 plasmids in *E. coli* ST940, *K. pasteurii* ST416, and *K. pneumoniae* ST258 that harbored *bla*_NDM-5_, *qnrS1*, and *bla*_SHV_, respectively.

This study has some limitations. First, the study design focused on MGEs in isolates from this specific locality, potentially overlooking other MGEs that may also have played a role in transmission. Second, metadata were unavailable for the patient isolates due to data protection concerns. These data would have facilitated a better comparison of patient isolates and allowed a more precise temporal comparison of patient and environmental isolates. Finally, due to resource constraints, the conjugation study was only carried out at one temperature (37°C) and only tested in one recipient (*E. coli*). As such, it does not consider the variability of conjugation at different temperatures and in different species, which could be particularly important in the context of environmental transmission dynamics (54).

### Conclusion

The detection of highly similar plasmids and MGEs bearing *bla*_OXA-48_, *bla*_KPC-2_, and *bla*_NDM-5_ genes in different species and STs illustrates their key role in the local dissemination of these CEGs between Enterobacterales from human and environmental niches. It is not possible based on this study to discern the directionality of their movement, but it is likely to be bidirectional. The use of long read or hybrid WGS for CPE surveillance and epidemiology facilitates the detailed characterization of these MGEs and plasmids. This understanding will support the development of strategies for early containment of such elements and minimize human exposure.

## Data Availability

Hybrid assemblies of all isolates were uploaded to NCBI under BioProject number PRJNA1064408. Individual accession numbers are listed in Table S5 in the supplemental material.

## SUPPLEMENTAL MATERIAL

Supplemental tables and figures spectrum.00904-24-s0001.xlsx Tables S1 to S6; Fig. S1 to S3.

ASM does not own the copyrights to Supplemental Material that may be linked to, or accessed through, an article. The authors have granted ASM a non-exclusive, world-wide license to publish the Supplemental Material files. Please contact the corresponding author directly for reuse.
